# Characterization of a sperm motility signalling pathway in a gonochoric coral suggests conservation across cnidarian sexual systems

**DOI:** 10.1098/rspb.2023.0085

**Published:** 2023-08-09

**Authors:** Benjamin H. Glass, Jill Ashey, Amarachukwu R. Okongwu, Hollie M. Putnam, Katie L. Barott

**Affiliations:** ^1^ Department of Biology, University of Pennsylvania, Philadelphia, PA 19104, USA; ^2^ Department of Biological Sciences, University of Rhode Island, Kingston, RI 02881, USA

**Keywords:** coral reproduction, sperm physiology, cell signalling, soluble adenylyl cyclase, sexual systems, coral sperm

## Abstract

Most stony corals liberate their gametes into the water column via broadcast spawning, where fertilization hinges upon the activation of directional sperm motility. Sperm from gonochoric and hermaphroditic corals display distinct morphological and molecular phenotypes, yet it is unknown whether the signalling pathways controlling sperm motility are also distinct between these sexual systems. Here, we addressed this knowledge gap using the gonochoric, broadcast spawning coral *Astrangia poculata*. We found that cytosolic alkalinization of sperm activates the pH-sensing enzyme soluble adenylyl cyclase (sAC), which is required for motility. Additionally, we demonstrate for the first time in any cnidarian that sAC activity leads to protein kinase A (PKA) activation, and that PKA activity contributes to sperm motility activation. Ultrastructures of *A. poculata* sperm displayed morphological homology with other gonochoric cnidarians, and sAC exhibited broad structural and functional conservation across this phylum. These results indicate a conserved role for pH-dependent sAC-cAMP-PKA signalling in sperm motility across coral sexual systems, and suggest that the role of this pathway in sperm motility may be ancestral in metazoans. Finally, the dynamics of this pH-sensitive pathway may play a critical role in determining the sensitivity of marine invertebrate reproduction to anthropogenic ocean acidification.

## 1. Introduction

Sexual reproduction is ubiquitous across the animal tree of life, and species have developed diverse mechanisms for the allocation of sexual cell types among individuals (i.e. sexual systems) [1]. The phylum Cnidaria comprises a diverse assemblage of species whose sexual systems are not always correlated with their phylogenetic relationships [2]. For example, there are both gonochoric and hermaphroditic species within the cnidarian orders Scleractinia (stony corals) and Actiniaria (sea anemones), and even congeneric species can display different sexual systems [3–5]. Cnidarians also display variation in reproductive strategies, with some species exhibiting internal fertilization and brooding of larvae within the maternal polyp, while others liberate sperm and eggs into the water column for external fertilization. In broadcast spawning, sperm perform taxis towards an egg for fertilization [6]. While the sexual systems and reproductive strategies of many cnidarian species have been described [3], we lack a thorough understanding of cnidarian sperm biology, which limits conservation strategies to protect these ecologically important species from the effects of anthropogenic climate change [7].

Early work investigating cnidarian sperm revealed a diversity of morphologies [8–18], and showed that sperm could be categorized into distinct morphological ‘types’, which were found to be tightly associated with sexual system in addition to differing within and between phylogenetic groups [8–10]. Specifically, sperm from hermaphroditic cnidarians tend to display rounded nuclei, whereas sperm from gonochoric species typically have conically shaped nuclei [11]. Additionally, sperm from gonochoric cnidarians show partial fusion of mitochondria, the presence of dense perinuclear vesicles, and a dense lipid body, whereas these features are absent in sperm from hermaphroditic species [11]. As sperm morphology is associated with function across phyla [19], these differences in morphology are likely associated with different sperm functional constraints. Indeed, sperm from hermaphroditic corals appear to have receptors for egg-derived immobilizing factors (in addition to chemoattractants), which have never been described in gonochoric species [20–23], indicating that sperm molecular phenotypes may differ between coral sexual systems. Yet, it remains unknown whether sperm from gonochoric and hermaphroditic corals differ in terms of the molecular mechanisms that regulate their motility, a central function necessary for successful reproduction.

Recent work has shown that components of a molecular sperm motility signalling pathway involving the enzymes soluble adenylyl cyclase (sAC) and protein kinase A (PKA) are conserved between sea urchins and the hermaphroditic coral *Montipora capitata* [24]. This ‘sAC-cAMP-PKA’ signalling pathway has been most thoroughly described in sea urchin sperm, where the signalling cascade begins with the binding of an egg-derived substance (e.g. speract) to guanylyl cyclase A (GC-A; also known as speract receptor), a sperm transmembrane receptor [24–26]. The activation of GC-A results in the production of the second messenger cyclic guanosine monophosphate (cGMP), which activates the potassium-selective, cyclic nucleotide gated channel (CNGK), resulting in the removal of H^+^ ions from the sperm cytosol via the Na^+^–H^+^ exchanger SLC9C1 [20,27]. This leads to an increase in sperm intracellular pH (pH_i_), which activates the pH-sensing enzyme sAC [28]. Activated sAC then converts adenosine triphosphate (ATP) to cyclic adenosine monophosphate (cAMP), a second messenger that modulates the activity of PKA and other downstream targets to produce sperm motility [28]. Homologues of each component of the sea urchin pathway are expressed in sperm from the hermaphroditic coral *M. capitata*, and sperm cytosolic alkalinization leads to the activation of adenylyl cyclases (ACs) and PKA, followed by the onset of sperm motility in this species [24]. However, several aspects of this pathway remain undescribed in cnidarians. For example, it is still unknown whether AC activity during the onset of motility is due to sAC (as opposed to transmembrane ACs), whether sAC activity is necessary for activating PKA, or if PKA itself is necessary for sperm motility. It is also unknown whether some or all components of this pathway are conserved in gonochoric corals, which may be unlikely given the morphological and molecular differences between sperm from gonochoric and hermaphroditic species. Here, we address this knowledge gap by employing a variety of molecular techniques to characterize the role of sAC and PKA in regulating sperm motility in the gonochoric coral *Astrangia poculata*. We then performed an analysis of published proteomes to investigate whether sAC displays structural and functional conservation across cnidarian species with diverse sexual systems.

## 2. Materials and methods

### (a) Coral collection and spawning

**Figure 1.**
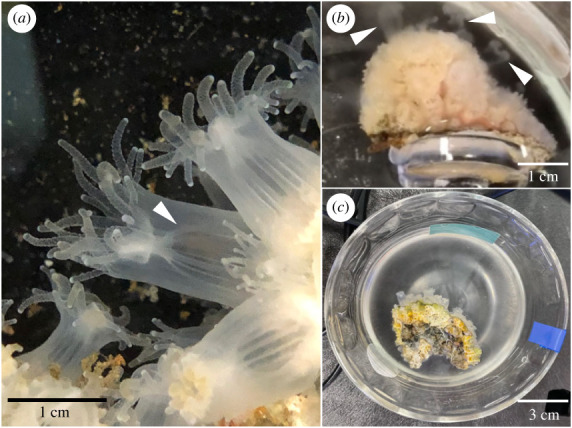
*Astrangia poculata* colony and spawning images. (*a*) A close-up image of several polyps of *Astrangia poculata* in its aposymbiotic form. White arrowhead indicates one polyp's mesenteries (the site of gametogenesis). (*b*) An aposymbiotic, male colony of *A. poculata* actively spawning sperm, which are seen being released from the polyps in concentrated bursts (white arrowheads). (*c*) An aposymbiotic, male colony of *A. poculata* sitting in seawater cloudy with recently spawned sperm. Scale bars are included in all images.

Adult *Astrangia poculata* (Ellis & Solander, 1786) colonies (figure 1*a*) were collected from Narragansett Bay, RI, USA (41.49231, −71.41883) in early August 2021 and 2022 and transported in seawater to the University of Rhode Island. Corals were induced to spawn by increasing the temperature from approximately 22°C to 31°C over 30 min and physical touch. Once spawning began (figure 1*b*), males were moved to individual glass bowls containing 50 ml seawater and left unperturbed until spawning activity ceased (figure 1*c*). The seawater containing sperm (i.e. ‘sperm water’) was then passed through a 100 μm cell strainer. For *in vivo* assays, sperm from each male were concentrated separately by centrifugation at 1500 × *g* for 5 min. The supernatant was removed, additional sperm water from the same male was added atop the sperm pellet, and centrifugation was repeated to form a large sperm pellet. Following this process, the supernatant was removed and sperm were resuspended in approximately 15 ml sodium-free seawater (NaFSW)—a seawater substitute with the same salinity as seawater but lacking sodium ions (Na^+^) [22,24,29]. The absence of Na^+^ in NaFSW prevents the outward flux of H^+^ through an Na^+^/H^+^ exchanger and thus facilitates cytosolic alkalinization following the addition of NH_4_Cl [22,24,29]. Sperm in NaFSW were used for all *in vivo* assays within 2.5 h after spawning. Since coral sperm capacity for motility can decline over time following spawning [30], each pool of sperm in NaFSW was tested for motility capacity immediately prior to use in each *in vivo* assay by stimulating a small aliquot of the pool with 20 mM NH_4_Cl; all sperm pools had at least 50% motility as assessed via phase contrast microscopy (Nikon Eclipse Ni-U microscope and digital camera system DS-Ri1).

### (b) Transmission electron microscopy

**Figure 2.**
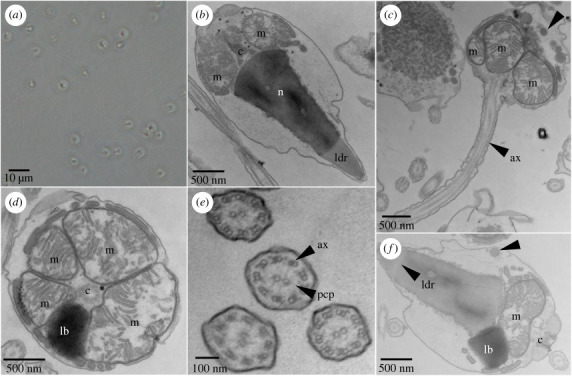
*Astrangia poculata* sperm ultrastructure. (*a*) A brightfield image of multiple sperm. (*b–f*) Transmission electron micrographs of *Astrangia poculata* sperm. (*b*) A sagittal view of the internal structure of a single sperm head. Labelled structures include the region of low density (ldr) at the anterior tip of the nucleus (n), two mitochondria (m), and the centriolar complex (c). (*c*) A parasagittal view of a sperm cell depicting the base of the head leading into the tail. Labelled structures include the flagellar axoneme (ax), three mitochondria (m), and perinuclear vesicles (e.g. black arrowhead). (*d*) A transverse view of the base of a sperm head depicting four mitochondria (m), a dense lipid body (lb), and the centriolar complex (c). (*e*) A transverse view of several sperm flagella depicting axonemes (ax) with their classical 9 + 2 microtubule arrangement. Pericentriolar processes (pcp) are somewhat visible between the central microtubule pairs and the outer microtubule doublets of each flagellum. (*f*) A sagittal view of the internal structure of a sperm head, again depicting the anterior region of lower density (ldr), dense lipid body (lb), mitochondrion (m), centriolar complex (c), and vesicles (black arrowhead).

Sperm in seawater were pelleted as described above until the pellet was approximately 100 µl, at which point the supernatant was removed and the pellet was fixed with 2.5% (v/v) glutaraldehyde and 2.0% (v/v) paraformaldehyde in 0.1 M sodium cacodylate buffer (pH 7.4) overnight at 4°C. After subsequent buffer washes, the sample was post-fixed in 2.0% (w/v) osmium tetroxide with 1.5% (w/v) K_3_Fe(CN)_6_ for 1 h at room temperature, and rinsed in deionized (DI) H_2_O prior to *en bloc* staining with 2% (w/v) uranyl acetate. After dehydration through a graded ethanol series, the sample was infiltrated and embedded in EMbed-812 (Electron Microscopy Sciences). Thin sections were stained with uranyl acetate and SATO lead, then examined with a JEM-1010 electron microscope (JEOL) fitted with a Hamamatsu digital camera and AMT Advantage NanoSprint500 software (figure 2).

### (c) Western blotting for sAC and PKA

Western blots were performed to examine sAC and PKA protein expression in sperm. Briefly, sperm pellets from individual males (*N* = 11) were flash frozen, lysed, sonicated and their protein concentrations determined via a Bradford assay. Next, 1.08 µg (sAC, Aug. 2021) or 1.03 µg (sAC and PKA, Aug. 2022) protein were separated in a 4–12% tris-glycine gel followed by transfer to a polyvinylidene fluoride (PVDF) membrane, blocking, and incubation with custom polyclonal primary antibodies against coral sAC (GenScript [31]), custom sAC antibodies preabsorbed with sAC peptide, or commercial antibodies against PKA (Cell Signaling Technology). Finally, the membranes were treated with a secondary antibody (anti-rabbit IgG with horseradish peroxidase) and imaged via chemiluminescence. Each blot was also probed for β-tubulin as a loading control. Additional details are provided in the electronic supplementary material.

### (d) Immunocytofluorescence imaging of sAC in sperm

Immunocytofluorescence was performed to determine the localization of sAC in *A. poculata* sperm. Briefly, sperm were fixed in 4% (v/v) paraformaldehyde and incubated on a glass slide with either primary antibodies (0.55 µg ml^−1^ anti-sAC and 2.5 µg ml^−1^ anti-β-tubulin) diluted in blocking buffer or blocking buffer alone (controls). Next, sperm were incubated with secondary antibodies (anti-rabbit IgG AlexaFluor 488 and anti-mouse IgG AlexaFluor 594 at 4 µg ml^−1^; Abcam), sealed under a glass coverslip with mountant containing DAPI, and imaged on a Leica DMi8 confocal microscope. Details are provided in the electronic supplementary material.

### (e) Assessment of *in vivo* sAC activity

Within 0.5–1.5 h post-spawning, each pool of sperm in NaFSW (*N* = 3 pools; 1 pool per male) was divided in half and incubated with either dimethyl sulfoxide (DMSO; 0.5% v/v) as a carrier control or the sAC inhibitor KH7 (50 µM in DMSO; Tocris Bioscience) for 30 min, then aliquoted into a 96-well plate. For each sperm pool, half of the replicate wells of sperm from each treatment (DMSO or KH7) were amended with either 100 mM NH_4_Cl to a final concentration of 20 mM NH_4_Cl (stimulated) or an equivalent volume of NaFSW (unstimulated). Triplicate wells per treatment and stimulation condition were lysed at time = 0, 0.1, 0.5, 1, 2 and 5 min with the addition of HCl (0.167 M final concentration) with 0.01% (v/v) Triton-X. Cyclic adenosine monophosphate (cAMP) was quantified in each sample well using an enzyme-linked immunosorbent assay according to manufacturer's instructions (ArborAssays K019). Data were analysed using a four parameter logistic curve of cAMP standards to determine the nmol of cAMP in each well, which was normalized to ng of protein in the well determined via Bradford assays.

### (f) Prediction of PKA C*α* three-dimensional structures

A preliminary *A. poculata* proteome produced by Guiglielmoni [32] using whole adult tissue was queried for expression of PKA C*α*. Specifically, a custom protein basic local alignment search tool (BLASTp) database was created from a FASTA file containing the *A. poculata* proteome [32]. Searching the database using the epitope against which the commercial PKA C*α* antibody was designed (KKGAKNDIIKAFLTEAK) yielded two highly similar protein results, which were confirmed to be isoforms of PKA C*α* via alignment of their amino acid sequences against human PKA C*α* with Clustal Omega [33]. Next, predicted molecular weights were calculated for each isoform by adding the individual molecular weights of their constituent amino acids. Finally, three-dimensional structures were generated with AlphaFold [34] and visualized with Mol* [35].

### (g) Assessment of *in vivo* PKA activity

**Figure 3.**
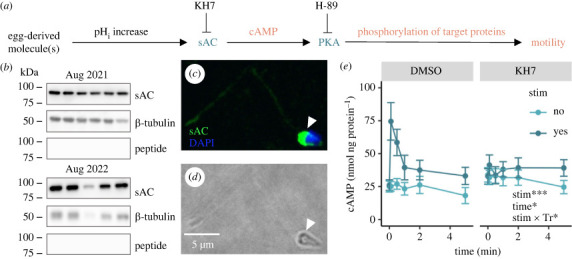
Sperm motility pathway, experimental design, and expression, localization, and activation dynamics of soluble adenylyl cyclase (sAC) in *Astrangia poculata* sperm. (*a*) Simplified schematic of the sea urchin sperm motility pathway (black), with text in colour indicating Western blots (blue) and *in vivo* assays (orange) performed to probe aspects of the pathway in *Astrangia poculata* sperm. Inhibitors used in *in vivo* assays (KH7 and H-89) point to their targets with inhibition arrows. (*b*) Western blots for sAC, β-tubulin (loading control), and sAC again following antibody–peptide preabsorption (nonspecific binding control) in sperm samples across two spawning seasons (Aug. 2021 and 2022). Each lane represents sperm from a distinct male colony (*N* = 11 males). (*c*) Representative immunocytofluorescence micrograph of a single sperm cell. Labels indicate staining for sAC (green) and DNA (DAPI; blue). White arrowhead indicates sAC staining at the base of the sperm head. (*d*) Brightfield image corresponding to image in (*c*); scale bar applies to both images. (*e*) Sperm *in vivo* cyclic adenosine monophosphate (cAMP) concentration over time following incubation with DMSO or the sAC inhibitor KH7 and in the presence or absence of 20 mM NH_4_Cl stimulation (stim), which was administered immediately after time = 0 min. Each point represents the average of cAMP concentrations for separate pools of sperm from three distinct male colonies, which were each assessed in triplicate as technical replicates for a total of *N* = 9 assays per point. Bars represent the standard error of the mean, and the inset identifies stim, time, and the interaction between stim and drug treatment (Tr) as significant linear model terms (Type III ANOVA; *p less than 0.05, ***p less than 0.001).

PKA activity was assessed *in vivo* to test the hypothesis that PKA is activated downstream of sAC following cytosolic alkalinization in sperm from *A. poculata* (figure 3*a*). Within 1.5–2 h post-spawning, pools of sperm in NaFSW (N = 3 males; 1 pool per male) were divided into three aliquots and treated with either DMSO (0.5% v/v), the sAC inhibitor KH7 (50 μM in DMSO), or the PKA inhibitor H-89 (20 µM in DMSO; Cell Signaling Technologies) for 30 min. Sperm from each treatment were then aliquoted into each of six 1.5 ml tubes. One tube from each treatment was flash frozen after addition of NaFSW as an unstimulated control. Then, each of the remaining tubes was stimulated with the addition of a stock solution of 100 mM NH_4_Cl (20 mM NH_4_Cl final concentration). One tube per treatment was then flash frozen in liquid nitrogen at either 0.1, 0.5, 1, 2, or 5 min post-stimulation. Later, proteins were extracted from each sample, quantified via Bradford assays, and run in gel electrophoresis as described above. Following transfer, membranes were probed with a commercial primary antibody recognizing phosphorylated substrates of PKA (electronic supplementary material, figure S1C; Abcam). All blots were developed with pico chemiluminescence reagents (Thermo Fisher Scientific), and imaged on an Amersham Imager 600 (General Electric) with automatic exposure. Total band intensity was quantified for each lane and normalized to the time = 0 lane for each blot in ImageJ [36]. Additional details are presented in the electronic supplementary material.

### (h) Assessment of sperm motility

Sperm motility was assessed within 0.5–2.5 h of spawning under various conditions to determine the roles of sAC and PKA in motility (figure 3*a*). Videos of sperm in seawater or NaFSW were recorded before and immediately after stimulation with 20 mM NH_4_Cl. For videos of sperm in seawater, sperm were never transferred to NaFSW and sat in seawater for up to 2.5 h, so motility results of sperm in seawater should be interpreted with caution as motility may have declined over time. Next, sperm in NaFSW were amended with either DMSO (0.05% v/v), KH7 (50 µM in DMSO), or H-89 (20 µM in DMSO) and incubated for 30 min, and then stimulated with 20 mM NH_4_Cl and immediately videoed under a phase contrast microscope (Nikon Eclipse Ni-U microscope and digital camera system DS-Ri1). This experiment was repeated using sperm from each of three males. In all movies, most non-motile sperm could be seen twitching, indicating viability [24]. Each movie was renamed with a random string of characters and corresponding treatment information was recorded in a spreadsheet, then movies were scored by an observer blinded to the treatment as 0, 20, 40, 60, 80, or 100% motile according to the percentage of sperm displaying progressive, directional motility. Motility scores for each treatment were averaged for each male (technical replicates) and then across the males (biological replicates). Details are located in the electronic supplementary material.

### (i) Investigation of *A. poculata* proteome for sperm motility pathway homologues

The proteome of *A. poculata* [32] was investigated via local BLASTp for the presence of homologues to proteins making up the canonical sperm motility pathway in other marine invertebrate species, including sea urchins and the coral *Montipora capitata* [24]. These proteins are GC-A, the potassium-selective CNGK, the hyperpolarization-activated, cyclic nucleotide-gated channel (HCN), the four subunits of CatSper and the sperm-specific Na^+^/H^+^ exchanger (sNHE). When a definitive homologue (E-value = 0.0) was detected, sequences were counted for length in amino acid residues, then the molecular weights were calculated as described above. A summary of query sequences, sources, and E-values for this analysis is provided in electronic supplementary material, table S1.

### (j) Cnidarian sAC sequence alignment, domain prediction and phylogenetic tree generation

Genome, transcriptome and proteome data were investigated for the presence of sAC homologues for 16 cnidarian species representing six of the major clades (octocorals, sea anemones, corallimorphs, stony corals, box jellyfish and true jellyfish). For each species, sAC homologues were identified through either BLAST or ORTHOSCOPE [37], then the resulting sequences were subjected to domain prediction with InterPro [38] and multiple sequence alignment (MSA) generated via seeded guide trees and hidden Markov model profile–profile techniques with Clustal Omega [33]. A graphic of the MSA was produced in Jalview v. 2.11.2.5 [39], and Clustal Omega was also used to generate a phylogenetic tree based on the sAC amino acid sequences via neighbour joining without distance correction. Additional information is located in the electronic supplementary material.

### (k) Data analysis

All statistical analyses and plot generation were performed using RStudio version 2022.7.1.554 (RStudio Team 2023). For statistical analysis of data from *in vivo* assays, linear models, linear mixed effect models and generalized additive models were created relating response variables (e.g. normalized contractions of cAMP) to the factors time, treatment (DMSO, KH7, or H-89), and stim (presence or absence of 20 mM NH_4_Cl) individually and in each possible additive and interactive combination (e.g. time × treatment or time + treatment). Models were compared for goodness of fit using the Akaike information criterion (AIC) and the best model was subjected to an ANOVA (Type II or III) followed by Tukey's honest significant difference tests for pairwise comparisons. Additional details are in the electronic supplementary material.

## 3. Results

### (a) Sperm ultrastructural characterization via transmission electron microscopy

Transmission electron microscopy (TEM) was used to characterize the ultrastructure of *Astrangia poculata* sperm (*N* = 3 males; figure 1*a–c*). TEM micrographs showed that sperm had a head size of approximately 2.5 µm (greatest width) by 3 µm (greatest length), and displayed a tail length of approximately 45 µm (figure 2*a*). At the base of the sperm head, 2–4 mitochondria formed a semicircular structure around the centriolar complex, which gave way to the flagellar axoneme at the base of the head (figure 2*b–f*). An electron dense lipid body was located planar with the mitochondria; together, these structures formed a full ring around the base of the sperm head, which filled the cytoplasmic space from the outer plasma membrane to the small centriolar complex (figure 2*d*,*f*). A transverse section of the flagellar axoneme revealed microtubules in the classical 9 + 2 arrangement, and pericentriolar processes could be faintly seen connecting the central microtubule pair to the outer microtubule doublets (figure 2*e*). The nucleus was conically shaped, located anterior to the mitochondria and lipid body, and included a region of low electron density at the anterior tip (figure 2*b*,*f*). The tip of the nucleus was directly adjacent to the outer plasma membrane; in contrast, more cytoplasmic space existed between the nucleus and plasma membrane near the nuclear base (figure 2*b*,*f*). This space was mostly devoid of any visible structures, but some electron dense vesicles were observed in this region (figure 2*f*), as well as in the space between the mitochondria and the outer membrane (figure 2*b–d*,*f*).

### (b) Expression, localization and activity of sAC in sperm

We set out to characterize aspects of the sea urchin sperm motility pathway (figure 3*a*) in *A. poculata* sperm. Western blotting for sAC in *A. poculata* sperm revealed expression of a single major isoform approximately 88 kDa in weight (*N* = 11 males; figure 3*b*). Immunocytofluorescence revealed that sAC was localized most abundantly at the base of the sperm head, and was also present in low abundance throughout the sperm tail (*N* = 4 males; figure 3*c*,*d*; electronic supplementary material, figure S1A–B). Baseline levels of cAMP were 25 ± 4 nmol cAMP per ng protein in DMSO treated sperm (figure 3*e*). Addition of 20 mM NH_4_Cl, which induces cytosolic alkalinization, resulted in a 3-fold increase in cAMP content within six seconds, which peaked at 75 ± 14 nmol cAMP per ng protein. This level began to decrease by 30 s post-stimulation, returning to just above the baseline at 33 ± 7 nmol cAMP per ng protein by five minutes (figure 3*e*). By contrast, in the presence of the sAC inhibitor KH7, there was no increase in cAMP concentration at six seconds following stimulation with 20 mM NH_4_Cl, and cAMP levels remained near the baseline for five minutes post-stimulation (figure 3*e*). Stimulation with NH_4_Cl (Type III ANOVA; *p* < 0.001), time (*p* < 0.001), and the interaction between treatment and stimulation (*p* < 0.05) were significant predictors of sperm cAMP levels (*N* = 3 males; 3 assays per male per treatment).

### (c) Structure, expression and activity of PKA in sperm

**Figure 4.**
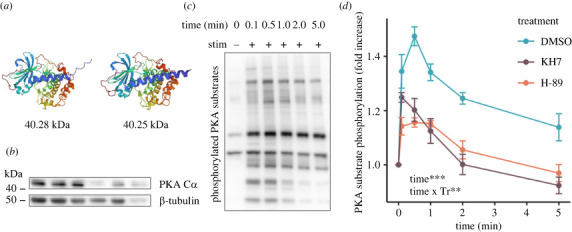
Structure, expression, and activation dynamics of protein kinase A (PKA) C*α* in *Astrangia poculata* sperm. (*a*) Predicted three-dimensional structures for two isoforms of protein kinase A (PKA) C*α* identified from the *Astrangia poculata* proteome, with their corresponding predicted molecular weights. (*b*) Western blot for PKA C*α* displaying two bands just above 40 kDa, which are assumed to be the isoforms of PKA C*α* depicted in (*a*). Blot for β-tubulin is also shown as a loading control. Each lane represents sperm from a different male colony (*N* = 6 males). (*c*) Representative Western blot for phosphorylated PKA substrates of a single pool of sperm treated with a DMSO carrier control over time following stimulation with 20 mM NH_4_Cl (stim), which was administered directly after time = 0 min. (*d*) Quantification of phosphorylation of PKA substrates over time following sperm stimulation with 20 mM NH_4_Cl, expressed as a fold increase in the intensity of bands from Western blots for phosphorylated PKA substrates. Each point represents the average fold increase relative to unstimulated conditions for sperm treated with DMSO (negative control), the sAC inhibitor KH7 (50 µM in DMSO), or the PKA inhibitor H-89 (20 µM in DMSO). Sperm from three different male colonies were assessed for each treatment. Inset identifies time and the interaction between time and treatment (Tr) as significant linear model terms (Type III ANOVA; **p < 0.01, ***p < 0.001), and bars represent the standard error of the mean.

We identified two isoforms of the C*α* subunit of PKA, which differed from each other in a few C-terminal amino acids and were estimated to be 40.28 and 40.25 kDa in weight, respectively. Generation of three-dimensional structures for both PKA C*α* isoforms via AlphaFold [34] showed that the two proteins were nearly identical in structure, with the lighter isoform lacking a small helix at the C-terminus compared to the heavier isoform (figure 4*a*). Western blots showed that *A. poculata* sperm expressed both isoforms of PKA C*α*, with the lighter isoform displaying slightly higher expression compared to its heavier counterpart (*N* = 6 males; figure 4*b*). In sperm treated with DMSO as a carrier control, stimulation with 20 mM NH_4_Cl resulted in increased PKA activity within six seconds, as evidenced by an increased number and darkening of bands on a Western blot treated with antibodies detecting phosphorylated PKA substrates (figure 4*c*). Quantification of the cumulative Western blot band intensity indicated that PKA activity increased rapidly following stimulation with 20 mM NH_4_Cl, peaking at 1.47 ± 0.04 times baseline levels by 30 s (figure 4*d*; electronic supplementary material, figure S1C–D). Levels of PKA substrate phosphorylation remained elevated at around 1.14 ± 0.05 times baseline levels for at least five minutes post-stimulation for sperm treated with DMSO (figure 4*d*). Incubation of sperm with the sAC inhibitor KH7 prior to stimulation changed the kinetics and intensity of PKA activation. Specifically, sperm treated with KH7 showed a 1.2-fold smaller peak in PKA substrate phosphorylation relative to controls of only 1.25 ± 0.02 times baseline levels, which was observed at six seconds following stimulation, as opposed to the peak at 30 s observed in sperm treated with DMSO (figure 4*d*). Furthermore, levels of PKA substrate phosphorylation decreased back to baseline (1.00 ± 0.04) by two minutes following stimulation in KH7 treated sperm, and were below baseline levels (0.92 ± 0.03) at five minutes (figure 4*d*). When treated with the PKA inhibitor H-89 prior to stimulation, sperm displayed 1.3-fold lower PKA substrate phosphorylation activity relative to DMSO controls, with a peak at 1.16 ± 0.02 times baseline levels occurring at 30 s before a return to just below baseline (0.96 ± 0.03) by five minutes (figure 4*d*). Time (Type III ANOVA; *p* < 0.05) and the interaction between time and treatment (*p* < 0.001) displayed statistically significant relationships with PKA substrate phosphorylation (*N* = 3 males; 1 assay per male per treatment and time combination).

### (d) Sperm motility under various conditions

**Figure 5.**
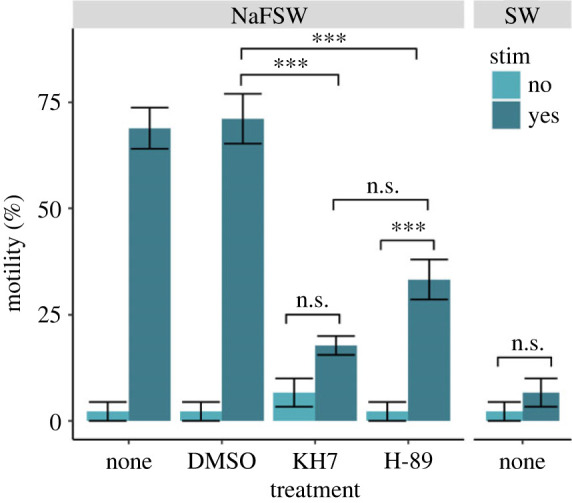
Motility of *Astrangia poculata* sperm under various treatment conditions. Average percent motility observed in movies of sperm swimming in either sodium-free seawater (NaFSW) or regular seawater (SW), and in the presence or absence of 20 mM NH_4_Cl stimulation (stim). Sperm were either untreated (none) or treated with DMSO (carrier control; 0.05% v/v), the sAC inhibitor KH7 (50 μM in DMSO), or the PKA inhibitor H-89 (20 µM in DMSO) prior to recording. Each bar represents an average of three movies (technical replicates) recorded for sperm from each of three distinct male colonies, resulting in *N* = 9 movies per treatment. Horizontal lines depict significance of pairwise comparisons (Tukey's HSD; n.s. = not significant, ***p < 0.001), while vertical error bars indicate the standard error of the mean.

Sperm in seawater (cell density range 1–5 × 10^6^ cells ml^−1^) exhibited no change in motility after stimulation with 20 mM NH_4_Cl (Tukey's HSD; *p* = 0.284), as was expected. For sperm in NaFSW, stimulation with 20 mM NH_4_Cl resulted in a significant increase in motility from a mean (± SEM) of 2 ± 2% to 69 ± 5% (*p* < 0.001). A significant increase in motility was also observed for sperm in NaFSW treated with DMSO as a carrier control following stimulation (from 2 ± 2% to 71 ± 6%; *p* < 0.001). By contrast, sperm in NaFSW incubated with the sAC inhibitor KH7 did not show a significant increase in motility after NH_4_Cl stimulation (*p* = 0.421). Following incubation with the PKA inhibitor H-89, sperm in NaFSW displayed a significant increase in motility after NH_4_Cl stimulation (from 2 ± 2% to 33 ± 5%; *p* < 0.001). However, the percentage of motile sperm following NH_4_Cl stimulation was significantly lower for sperm in the H-89 treatments than that for those in the DMSO carrier control (*p* < 0.001). All sperm motility data are displayed in figure 5 (*N* = 3 males; 3 movies per male per treatment).

### (e) Sperm motility pathway expression in *A. poculata* proteome

Queries of the *A. poculata* adult proteome resulted in identification of homologues for all proteins constituting the sperm motility pathway in sea urchins and other invertebrates (guanylyl cyclase A, the potassium-selective, cyclic nucleotide gated channel, the hyperpolarization-activated, cyclic nucleotide-gated channel, CatSper *α*1–4, and the sperm-specific Na^+^/H^+^ exchanger). Each of these homologues was the only significant (E-value < 10^−5^) match for each protein (electronic supplementary material, table S1). A summary of the function of these proteins with the lengths and predicted molecular weights of the identified *A. poculata* homologues are presented in electronic supplementary material, table S2.

### (f) Cnidarian sAC protein multiple sequence alignment and phylogeny

**Figure 6.**
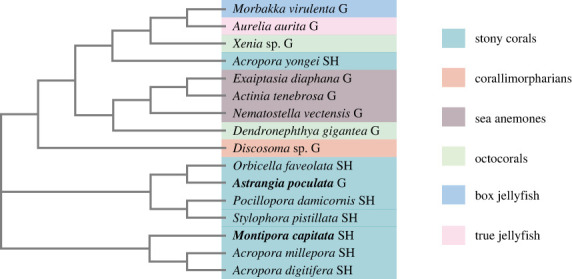
Phylogenetic tree of cnidarian sAC. Phylogenetic tree of 16 cnidarian species built using the amino acid sequences of sAC homologues (predicted sequences or proteome data). Labels next to each name indicate sexual system (G = gonochoric, SH = simultaneous hermaphrodite), and colours depict grouping of recognized clades, which are also labelled. The positions of *Astrangia poculata* and *Montipora capitata* are highlighted by bold text.

A MSA of cnidarian full-length sAC protein sequences generated via seeded guide trees and hidden Markov model profile–profile techniques showed high levels of conservation of residues across 14 of 16 representative cnidarian species, which were chosen to represent six major cnidarian clades (octocorals, sea anemones, corallimorphs, stony corals, box jellyfish, and true jellyfish). The soft coral *Xenia* sp. and true jellyfish *Aurelia aurita* expressed sAC homologues 1100–1700 amino acids shorter than the other species (electronic supplementary material, figure S2). The corals *Acropora digitifera* and *Montipora capitata* expressed sAC homologues with extended C-terminal domains 100–300 amino acids longer than the other species, while the corals *Stylophora pistillata* and *Orbicella faveolata* expressed homologues with C-terminal domains 100–500 amino acids shorter than the other corals (electronic supplementary material, figure S2). The sea anemone *Nematostella vectensis* displayed a small insertion around amino acids 1000–1187 not shared by other species in its sAC homologue, as did *S. pistillata* (around amino acids 1617–1878; electronic supplementary material, figure S2). The sAC homologues for 15 of the 16 species (barring only *Xenia* sp.) contained at least one predicted adenylate/guanylate cyclase catalytic domain (electronic supplementary material, table S3). The sAC homologue expressed by *Xenia* sp. contained one predicted domain, a tetraricopeptide (TRP) repeat, which was also present in the sAC homologues expressed by *Actinia tenebrosa*, *Acropora digitifera*, *Acropora millepora*, and *Morbakka virulenta* (electronic supplementary material, table S3). The sAC homologues for all species except *Xenia* sp., *A. aurita*, and *O. faveolata* had AAA ATPase domains (electronic supplementary material, table S3). A phylogenetic tree built via neighbour joining algorithms without distance correction from the sAC homologue sequences (figure 6) indicated that the 16 species clustered into three major groups, with one composed of complex stony corals (*A. millepora*, *A. digitifera* and *M. capitata*), a second composed of robust stony corals (*O. faveolata*, *A. poculata*, *Pocillopora damicornis* and *S. pistillata*), and a third containing the remainder of the species. Accepted phylogenetic relationships were not reflected by the tree; for example, *Acropora yongei* did not group mostly closely with the other stony corals, and the octocorals did not form a clade (figure 6). Finally, the gonochoric cnidarians *M. virulenta* [40], *A. aurita* [41], *Disocoma* sp. [42], *Xenia* sp. [43], *D. gigantea* [44], *E. diaphana* [45], *A. tenebrosa* [46], *N. vectensis* and *A. poculata* were present across the tree, as were the hermaphroditic species *A. millepora* [47], *A. digitifera* [21], *A. yongei* [48], *M. capitata* [24], *O. faveolata* [10], *P. damicornis* [12], and *S. pistillata* [49].

## 4. Discussion

Our investigation of sperm from the gonochoric coral *Astrangia poculata* revealed for the first time in any invertebrate that inhibition of the enzyme sAC in sperm abolishes the production of cAMP following cytosolic alkalinization. Furthermore, sAC activity was necessary for motility, with inhibition resulting in a lack of downstream sperm motility. We also found that inhibition of sAC in sperm reduced PKA activity, and that PKA was necessary for the full activation of motility in response to cytosolic alkalinization, demonstrating for the first time in any cnidarian that PKA is activated downstream of sAC and is important for activating sperm motility. Additionally, we present updated ultrastructures of *A. poculata* sperm, which clarify the number of mitochondria and presence of pericentriolar processes. Finally, we queried published cnidarian proteomes and found that sAC, a central signalling node in the sperm motility pathway, demonstrates structural and functional conservation across a diversity of cnidarian species.

### (a) Sperm from *A. poculata* displayed a morphology typical of gonochoric anthozoans

We examined sperm spawned by male *A. poculata* colonies via TEM, which revealed novel insights into ultrastructural features compared to the only prior ultrastructural analysis of sperm from this species published over 40 years ago [18]. For example, we found that sperm from *A. poculata* contained 2–4 mitochondria, whereas previous work suggested the presence of only a single mitochondrion [18]. Furthermore, pericentriolar processes could be seen in our micrographs connecting the central microtubule pair to the outer microtubule doublets in the flagellar axoneme of *A. poculata* sperm, which was not previously reported. Pericentriolar processes are also observed in sperm from other gonochoric anthozoans, and may function to provide structural support to the flagellum [8]. At a broader level, *A. poculata* sperm displayed all of the ultrastructure features that are characteristic of sperm from gonochoric anthozoans, including: (1) a conically shaped nucleus with a region of low density at the anterior tip (the latter of which may serve as an acrosome); (2) partial fusion of mitochondria resulting in differences in mitochondrial number between sperm cells; (3) the presence of dense perinuclear vesicles; and (4) a dense lipid body planar with the mitochondria [10,11]. Thus, *A. poculata* sperm display a distinct morphological ‘type’ shared with sperm from other gonochoric but not hermaphroditic anthozoans, the latter of which have rounded nuclei and discrete mitochondria, but lack lipid bodies and perinuclear vesicles [12]. As different sperm morphologies correlate with molecular and functional differences within and outside of Cnidaria [10,19,50], these results call into question whether cnidarians with different sexual systems use the same sperm motility signalling pathway.

### (b) The initiation of *A. poculata* sperm motility is controlled by the sAC-cAMP-PKA pathway

Given their morphological and molecular dissimilarities, we hypothesized that the molecular mechanisms underlying sperm motility would differ between hermaphroditic and gonochoric corals. This hypothesis was not supported, as the initiation of sperm motility in *A. poculata* was controlled by a cell signalling pathway involving sAC and PKA, which also controls the onset of sperm motility in the hermaphroditic coral *Montipora capitata* [24]. We found that, in sperm from *A. poculata*, a single isoform of sAC approximately 88 kDa in weight was expressed in the sperm head as well as throughout the flagellum—a localization that supports sAC's purported role in regulating flagellar beating, which was also observed in *M. capitata* [24]. Interestingly, the molecular weight of this protein did not match that of the predicted full-length sAC sequence in the *A. poculata* proteome (209.94 kDa), likely due to alternative splicing or post-translational modifications, which may be sperm-specific and are common for this protein across taxa. For example, in the coral *Pocillopora damicornis*, sAC is alternatively spliced and the major isoform expressed is a 94 kDa isoform containing only the two catalytic and P-loop domains (pdsAC_94_) [31]. We created an amino acid sequence for a hypothetical isoform of *A. poculata* sAC matching the domain structure of pdsAC_94_ and found that the resulting sequence has a predicted molecular weight of approximately 88 kDa, suggesting that we identified a major isoform of sAC in *A. poculata* homologous to that of *P. damicornis*, though this should be confirmed in future studies. Alternative splicing of sAC has been observed in cnidarians and humans [31,51,52], and the functions of different sAC isoforms could also be investigated in future studies.

Next, we found that sperm displayed a burst in cAMP production within six seconds following induced cytosolic alkalinization. Importantly, we showed for the first time in any invertebrate species that this cAMP spike was absent when sperm were pre-treated with the sAC inhibitor KH7, confirming that sAC activity is necessary for this response, as opposed to transmembrane adenylyl cyclases (tmACs). In addition, treatment with KH7 resulted in a significant decrease in motility activation compared to the DMSO control, confirming that sAC activity is necessary for motility in this species. PKA is typically activated downstream of sAC in other taxa [53], and here we found that sperm expressed two isoforms of PKA C*α* and displayed a rapid increase in PKA substrate phosphorylation following cytosolic alkalinization. PKA activity appeared to peak after the burst in cAMP production, suggesting that PKA is likely activated downstream of sAC in this species. In support of this hypothesis, we also found that PKA activity was depressed in the presence of the sAC inhibitor KH7, providing the first evidence, to our knowledge, that PKA activation occurs downstream of sAC in a cnidarian. Importantly, both PKA activity and sperm motility significantly decreased following treatment with the PKA inhibitor H-89, confirming for the first time that PKA activity is important for coral sperm motility, a mechanism that has also been observed in sea urchin sperm [27]. Interestingly, inhibition of PKA in sperm did not abolish all motility following stimulation, in contrast with the lack of motility following sAC inhibition, suggesting that sAC is particularly important for the initiation of motility, while there may be compensatory pathways for activating motility downstream of sAC when only PKA is inhibited. Together, these results demonstrate that induction of the sAC-cAMP-PKA signalling pathway activates sperm motility in *A. poculata*.

### (c) The *A. poculata* proteome contains homologues to the entire sea urchin sperm motility pathway

In sea urchins and *M. capitata*, sAC and PKA form part of a larger sperm motility pathway with several transmembrane proteins and other components [19,24]. We identified homologues of each of the known sperm motility pathway proteins—GC-A, the potassium-selective CNGK, HCN, CatSper *α*1–4, and sNHE—expressed in the adult proteome of *A. poculata*. Given the presence of the sperm-specific CatSper and sNHE proteins in a proteome generated from whole adult tissue from several animals, it is likely that one or more colonies contained developing spermatocytes [32]. Thus, we suggest that the entire sperm motility pathway operating in sea urchins and *M. capitata* is also present in *A. poculata*, indicating a high level of conservation across species with divergent sexual systems, as well as across phyla. This result has important evolutionary implications, as it suggests that the role of the sAC-cAMP-PKA pathway in controlling sperm motility may have arisen prior to the divergence of early branching metazoan phyla (e.g. Echinodermata and Cnidaria). However, presence of these proteins in the adult proteome does not necessarily equate to their presence in sperm, so this should be confirmed by future studies. Additionally, conservation of this pathway does not preclude differential signalling mechanisms between hermaphroditic and gonochoric species to initiate motility, and more work is needed to characterize the molecules that regulate the initiation of this pathway (e.g. types of ligands and receptors involved) and potential differences in sperm behaviour or performance (e.g. longevity, swimming pattern, etc.).

### (d) Cnidarian sAC homologues display broad structural and functional conservation

To further investigate the conservation of the sperm motility pathway across both gonochoric and hermaphroditic cnidarian species, we queried published genome, transcriptome, and proteome datasets for the presence of homologues to sAC, a central signalling node in this pathway, in 16 species representing six major cnidarian clades (octocorals, sea anemones, corallimorphs, stony corals, box jellyfish and true jellyfish). MSA of sAC homologues from these species showed a high degree of amino acid sequence similarity, which correlated with conservation of functional domains including adenylate cyclase and ATPase domains, suggesting that sAC-cAMP-PKA signalling is a mechanism shared by nearly all of these species that may be broadly involved in regulating sperm motility (as we show here for *A. poculata*). Interestingly, a phylogenetic tree of sAC amino acid sequences from these species showed clustering that was neither correlated with sexual system nor accepted phylogenetic relationships (e.g. lack of monophyly for the stony corals), demonstrating a possibly ancient origin for sAC in this phylum, which supports a conserved role for sAC in foundational biological processes such as sexual reproduction. Given that the activation of the sAC-cAMP-PKA pathway is stimulated by alkalinization of the sperm cytosol, this result also has important implications for the reproduction of broadcast spawning marine invertebrates in the face of anthropogenic ocean acidification, which is already known to alter sperm motility in many species after exposure of either sperm or adult males prior to spawning [6,54–59]. Future work should investigate the role of sAC-cAMP-PKA signalling in sperm from a broader diversity of species to clarify the mechanisms by which ocean acidification might impact reproduction across invertebrate phyla in future seas. For example, future research could investigate whether phosphodiesterase inhibitors and/or the addition of membrane-permeable cAMP analogues can rescue coral sperm motility under ocean acidification conditions.

### (e) The molecular sperm motility pathway is conserved across coral sexual systems

Overall, our study demonstrates that the molecular mechanisms underlying sperm motility in the hermaphroditic coral *M. capitata* also operate in the gonochoric coral *A. poculata*, suggesting an unexpected level of conservation of this pathway across sexual systems. We also uncovered broad structural and functional conservation of sAC, a central signalling node in the sperm motility pathway, across a diversity of both gonochoric and hermaphroditic cnidarian species. These results set the groundwork for future research investigating the role of sAC-cAMP-PKA signalling in sperm, while also providing insight into the evolution of the molecular mechanisms underlying sperm motility in an early branching metazoan phylum. Indeed, changes in sperm pH_i_ and concentrations of cyclic nucleotides are associated with sperm motility in hydrozoans, ascidians, sea cucumbers, starfish, sea anemones, corals, and humans [4,6,20–22,27,29], suggesting that sperm sAC-cAMP-PKA signalling may be widespread in metazoans. Future work investigating the sAC-cAMP-PKA pathway in sperm from other species would clarify the extent of its conservation. Finally, our work has important conservation implications for corals, which are facing the threat of global extinction due to anthropogenic climate change [7].

## Data Availability

The data and code are available from the Dryad Digital Repository: https://doi.org/10.5061/dryad.rn8pk0pg8 [60]. Supplementary material is available online [61].
